# Strategic changes and challenges of private dental clinics and practitioners in Israel: adapting to a competitive environment

**DOI:** 10.1186/s13584-024-00645-5

**Published:** 2024-10-01

**Authors:** Lior Naamati-Schneider

**Affiliations:** Health Systems Management Department, Hadassah Academic College, Jerusalem, Israel

**Keywords:** Dental healthcare market, Dental clinics, Strategic changes, Healthcare competitive market, Privet clinics

## Abstract

**Background:**

In the 21st century’s digital age, health organizations face challenges from reduced public healthcare spending, creating a competitive market that impacts healthcare management. The struggle is hardest for small organizations, such as private clinics. Competing under unfavorable conditions, these small businesses must run as independent, profitable units in a government-regulated market where they are subject to numerous restrictions yet receive no financial support. In a world of increasing dependence on digital technologies, these small businesses must adopt competitive business models and be adaptive and flexible in embracing change.

**Methods:**

This qualitative study is based on a thematic qualitative analysis of 20 in-depth, 45-minute-long interviews with dentists and owners of private dental clinics in various specialties. The study employs the strategic change model to examine how dentists who run private dental clinics implement new strategies and technologies to adjust to changes and create a competitive edge.

**Results:**

Six main categories emerged from the analysis of the interviews: *changes in the organization’s environment; instituting and assimilating changes; obstacles in embracing change; added value obtained from embracing the change; quality of care and service; and cost-benefit considerations*. The categories were map and evaluated in light of the strategic change model. The analysis indicated that digital strategies have been only partially adopted, suggesting an absence of a cohesive, long-term strategic vision for the organizations.

**Conclusions:**

The study explored the actions, perceptions, and challenges of adapting to a competitive digital market in dental private clinics. Based on these insights, recommendations have been provided for global change management, aiming for a sustainable and stable healthcare system that benefits the broader community.

## Introduction

The 21st century’s digital revolution has made evolving information technologies central to modern life [1, 2], adding pressure to global health systems already challenged by socio-political, demographic, economic, and regulatory factors [3]. These systems operate within a complex ecosystem influenced by multiple stakeholders, including policymakers, funding bodies, the public, and competitors [4]. The push to reduce public health spending [5, 6] creates a competitive and volatile environment, challenging healthcare management and stability [7, 8].

Historically, dental care in Israel was excluded from the National Health Insurance (NHI) law, leaving the majority of services to be funded privately. Recent reforms, which expanded public dental care for children and the elderly, have begun shifting the balance between private clinics and public providers, including HMOs, adding to the competition in the market [9, 10]. This unequal competitive environment, combined with the challenges of a changing ecosystem—such as adapting to the digital age, economic fluctuations, and demographic shifts—creates a pressing need for dental healthcare providers to implement new strategies. These changes are essential for clinics to survive and thrive in a rapidly evolving market [4, 8].

To stay competitive and deliver sustainable healthcare, organizations must implement strategic changes and embrace technology at all levels [11–14]. Large health organizations often manage this effectively [4, 5], but small, privately owned clinics, typically run by physicians without business training, often struggle with financial management and digital transformations [15].

This qualitative study examines how private dental clinics in Israel implement strategic changes to adapt to constant technological development. It aims to fill the research gap by exploring the transformation processes, perceptions, obstacles, and challenges faced by dentists in adapting to the changing market. Understanding this area will clarify processes, helping providers improve finances and offer sustainable services, benefiting the entire health system.

### Theoretical framework

Using Ginter et al.‘s strategic change model [8], this research explores how organizations manage change amid technological advances through three stages: strategic thinking, planning, and momentum. This process, involving all organizational levels, requires regular assessment and feedback, creating a semi-cyclic pattern of continuous adaptation.

The three stages of the model—thinking, planning, and execution—begin with s *trategic thinking stage*. This initial stage involves analyzing internal and external environments to recognize the need for organizational adjustment, requiring strong leadership to drive the change [16]. Following is the *strategic planning stage*, where insights are translated into actionable plans to achieve organizational objectives and fulfill its vision [8, 17]. This involves data collection, analysis, brainstorming, goal setting, and creating detailed action plans. Employee involvement at this stage enhances critical thinking and problem-solving [18]. The final stage, *strategic momentum*, involves executing the plan. This involves executing the plan and continuously evaluating and providing feedback. It integrates managing changes in external (regulation, competitors, technology) and internal factors (culture, employees, resources), requiring flexible and creative management to align planning and execution [8]. Kash et al. argue that organizations should transform their internal environments to support external adaptation by developing policies, establishing communication, and empowering employees [17].

To understand the adaptation patterns in the private health market, it is crucial to analyze the structure and regulatory aspects of the local dental and health market and the technological and economic shifts it faces. This analysis will clarify the context in which small clinics operate.

### The dental healthcare market: the case of Israel

Israel’s healthcare system integrates public universal coverage through HMOs with privately managed clinics [19, 20]. The public system covers alternative medicine and basic dental care for children and seniors. Recent reforms and environmental changes have shifted the balance between public and private sectors, intensifying competition. The general medical sector is increasingly privatized, facing uneven competition [4, 14, 19, 21]. Meanwhile, the dental sector is leaning towards public provision due to recent reforms and unbalanced competition from the public system [9, 10]. Dental services in Israel were historically excluded from the National Health Insurance (NHI) law, requiring private funding. Supplementary insurance was available, but high costs widened socio-economic disparities, with lower-income families often unable to afford care [22].

To address these disparities, a dental health reform was initiated in 2010, providing free preventive and low-cost restorative treatments for children up to age 8, with the program gradually extending to cover those up to age 18 by 2019. This reform also expanded to offer subsidized care for the elderly, starting with preventive and restorative treatments for those over 75, and including prosthetic treatments for those over 80. The aim to include individuals from age 67 onwards remains a long-term goal [9, 22–24]. Despite reforms, most dental care in Israel remains outside NHI coverage, with private spending making up 95% of national dental expenditure—7.7% of total healthcare expenses in 2016 [25] and 8.3% of GDP in 2020 [20]. Reforms for children and the elderly aim to improve access and outcomes, but challenges remain, particularly for the elderly. The 2019 reforms aimed to improve care for vulnerable groups, though socio-economic factors still impact oral health [26–28]. Research by Berg-Warman et al. [27] suggests that while oral health among Israel’s elderly has improved, further efforts are needed to meet global standards. Similarly, studies by Orenstein et al. [22] indicate that out-of-pocket dental care costs remain prohibitively high, calling for continued and expanded reforms to ensure equitable access.

However, despite the trend towards increased public provision (e.g., for children), the Israeli general dental market remains diverse and highly competitive. HMOs and large healthcare organizations now own and operate many dental practices, competing with private clinics, particularly in the children’s sector, but not exclusively. This competition is unbalanced due to differences in size, infrastructure and pricing abilities. It operates in a complex, partially managed market with limited regulation concerning quality of care, services, and competitive practices, while dealing with uneven competition from large healthcare organizations [14]. At the same time, a notable change driven by a consumer revolution has significantly increased public awareness and access to information, compelling healthcare organizations to adopt more customer-centric approaches. [29, 30]. The combination of these changes in the ecosystem and rising consumer demands contributes to intensifying competition. Clinics and practitioners face significant competitive pressures. This evolving landscape underscores the urgent need for continuous improvements in the quality of dental care, services, and competitive strategies to meet growing consumer demands and thrive in this unequal competitive environment. [14, 20–26].

### The era of technological advancements in healthcare and dental care

The global health market is experiencing a digital transformation, with technologies reshaping business practices and enhancing medical services for sustainable care [2, 11, 12, 31]. The COVID-19 pandemic in 2020 accelerated the adoption of remote medical services, driving rapid digital changes in health organizations, including dental medicine [32, 33]. According to Seif et al., [34], the dental profession’s perspective on how to best serve the entire patient journey must also expand to explore unprecedented opportunities to address critical choke points and unlock further value in global oral health. Even today, a typical day involves general dentists managing routine appointments with the aid of digital scheduling and diagnostic tools like digital radiography and intraoral cameras, enhancing patient communication and treatment accuracy. For example, Livny et al. (2024) presented a cross-sectional questionnaire-based study in Israel that examined the attitudes of parents regarding the use of dental apps. The conclusion was that health apps change interactions with healthcare professionals, enabling remote access to doctors and dentists. Parents of young children had positive perceptions of the usefulness of these applications as a tool to promote the oral health of their children [35].

Dental specialists utilize advanced technologies such as 3D imaging and digital scanning for complex procedures, improving precision and patient engagement. The adoption of electronic health records (EHRs), teledentistry, and AI-driven diagnostics streamlines operations and enhances care quality, underscoring digital transformation’s crucial role in modern dentistry. Moreover, innovations like computer-aided design/computer-aided manufacturing (CAD/CAM) and rapid prototyping (RP) further expand dental practice capabilities [36]. Augmented reality and virtual reality are used today in dentistry as technological tools for presenting digital visualizations and non-invasive simulations, which enable dentists to compare the expected results of different kinds of dental interventions and to offer personally fitted dentistry [37, 38]. In addition, a variety of advances have affected the clinics’ organizational and marketing services in the last decade, such as digitalization of information storage, visual imaging services, appointment scheduling, computerized reminders, and tele-dentistry, including remote diagnosis [37, 39, 40].

In recent years, Artificial Intelligence (AI) has profoundly transformed dentistry. The use of augmented intelligence (AuI) and AI-driven robotic systems like “Yomi” can improve efficiency and accuracy in clinical and administrative tasks. AI plays a crucial role in personalizing treatment plans, enhancing diagnostics through sophisticated image analysis, facilitating precise 3D printing of dental devices, and creating customized aesthetic solutions via Digital Smile Design. These advancements significantly improve dental care’s effectiveness and customization, making AI potentially indispensable in modern dentistry [41, 42]. Despite these advances, dental digitalization remains in its early stages with significant untapped potential. Current innovations primarily enhance care and pre-visit patient services like sales, marketing, and customer support [37]. Increasing digital literacy and greater reliance on mobile devices and the internet are likely to drive further advancements in online-based medical care [38]. However, there is still inconsistency in embracing these technologies and integrating them into clinics [37].

Digitalization requires careful planning, significant investment in equipment, and staff training. Larger organizations like hospitals and HMOs have an advantage due to their infrastructure and resources, rapidly adopting digital technologies, especially during the COVID-19 pandemic. While some were well-prepared, others had to adapt quickly [43]. In contrast, smaller private clinics face challenges due to differences in management, staff size, training, finances, and client volume. These factors have intensified competition and exacerbated disparities between the public and private sectors [44].

## The current research

### Method

This qualitative study is based on a thematic qualitative analysis of in-depth, 45-minute-long interviews with dentists in various specialties [45, 46]. The interviews took place between 2021 and 2023. The study has been approved by the ethics committee of the institution with which the author is affiliated. In-depth interviews give research participants an opportunity to express themselves freely using their own words and allowed the researchers to understand and map the range of obstacles and difficulties dentists deal with when adopting new strategies. Embracing change is often dependent upon the internal world of the dentist/manager; therefore, hearing their own descriptions of how they manage change provides insights into participants’ worldview and is thus critical to gaining a better understanding of the issue at hand [47].

### Research population

The study initially compiled a list exclusively comprising owners of private dental clinics. Utilizing the snowball sampling technique, we asked these dentists to recommend additional clinic owners, enhancing the diversity and breadth of our participant pool. This method guaranteed a varied representation of dental specialties from central Israel, comprising 20 participants aged between 35 and 60, with five women among them. All participants are owners of private dental clinics, and a small number work independently in clinics, making them the sole decision-makers regarding decisions and actions in their own clinics.

This collective expertise provides valuable insights into various aspects of managing private clinics. The participants selected cover a wide range of expertise, featuring 5 prosthodontists, 3 periodontists, 2 orthodontists, and 10 general dentists, ensuring a comprehensive perspective on the integration of technology in dental practices. More than half of the participants are experienced dentists with over 20 years of practice, while the remaining have 10 or more years of experience. They operate in multidisciplinary dental clinics that are not solely referral-based, and their clinics actively participate in the competitive market landscape.

The practitioners in this study come from diverse areas of dentistry, including general dentistry, offering a broad perspective. Notably, 8 participants, especially specialists, work in multiple clinics and public healthcare, such as hospitals or HMOs—a common practice in Israel [14, 20]. The clinics offer a wide range of services, with specialists collaborating closely. The selection focuses on multidisciplinary clinics, which are more likely to require advanced technological support due to the complexity of their services.

The research was conducted in the central region of Israel, which was chosen for its high level of competition in the dental industry, compared to peripheral regions. This geographic area has a high population density, with a large number of dental clinics operating in the area, leading to intense competition.

### Data collection tools

A semi-structured interview technique, featuring both open-ended questions and a pre-prepared interview guide with unseen broad questions, constituted the core data collection method. This format aimed to deeply explore healthcare professionals’ unique perspectives on technological advancements in healthcare and dentistry. The guide was intentionally designed to encourage discussions on specified subjects, including market changes, technological and digital transformations, and encountered challenges [48].

The interviews were conducted by the Principal Investigator (PI) and a team of research assistants who were students of health management. This team was carefully trained by the PI and received specific instruction to enhance their interviewing skills. This preparation included specific guidance aimed at equipping the assistants with the tools necessary to effectively process the data collected. This thorough training ensured a high level of consistency and sensitivity in data collection across all interviews. Upon completion of the interviews, the interviews were transcribed by the researcher and the trained team in accordance with strict transcription standards to ensure that the participants’ contributions were faithfully captured.

### Research procedure

Upon receiving ethical approval, we contacted candidates by email or phone. With their consent, we set either a face-to-face or an online Zoom interview. Participants were explained that their anonymity would be kept by avoiding publicity of any detail that could indicate identity or workplace, and that they were free to terminate the interview at any point [47, 49]. Participants were then presented with the topic of the research and asked general, open questions, to allow each interviewee to share information freely at their will [48]. A document with general questions was prepared in advance. This was not shown to the interviewee but formed the basis of a discussion with the interviewee. First, the interviewees’ perspectives on changes in dentistry and the healthcare market were explored. The participants were asked to: “Describe any recent changes you have experienced in the behaviour of the health care market in which your practice operates” and, if it hadn’t already been touched on, “please elaborate on the following”: Changes in the broader environment, oversight by the Ministry of Health, advances in technology and evolving needs, etc.” Participants were then asked to refer to changes in their practice or work environment. e.g., “Describe in your own words the organizational, business and medical changes that have taken place in your clinic – e.g.- technological, digital changes, different marketing, customer service, etc.” The next question was about the obstacles and challenges that accompanied these processes: e.g., “Describe in your own words your personal experiences, challenges and obstacles related to the processes described above”. The interviews focused on topics relevant to the study: long-term strategy, strategic changes and their implementation, digital transformation, marketing activities and competition in the dental market.

### Data analysis

The transcribed interviews underwent a detailed thematic analysis, where patterns and themes relevant to the study’s goals were identified and explored. This methodical analysis focused on uncovering insights related to strategic planning, digital transformation, and the competitive dynamics within the dental market, among other topics. By employing a thematic analysis, the study aimed to delve deeply into the strategic implications of technology integration within dental practices, offering a comprehensive view of how these entities are navigating the challenges and opportunities presented by technological and market developments. This analysis culminated in a nuanced understanding of the subject matter, aligning with the research objectives and contributing valuable perspectives on the adaptation strategies of dental practices in the face of ongoing technological evolution.

The interview data were analyzed in three stages. First, transcripts were read and re-read independently to identify themes, focusing on evaluating the study’s theoretical foundation and core objective: understanding processes of change [50]. Thematic analysis proved effective in revealing interviewees’ perspectives and shared views [51], with primary themes emerging at this stage. In the second stage, sub-categories were developed, organizing detailed themes and associations between levels to map perceptions. This iterative process continued until themes and categories accurately reflected participants’ feelings, supported by interview quotations [46, 51]. The themes highlighted strategic changes in private dental clinics. In the third stage, the findings were mapped and compared with the theoretical framework from Gintar et al. [8], which outlines structured change in healthcare. This comparison aimed to provide a comprehensive understanding of how participants perceive and manage ongoing changes.

## Results

Six main categories emerged from the analysis of the interviews: *changes in the dental clinic’s environment; instituting and assimilating changes; obstacles in embracing change; added value obtained from embracing the change; quality of care and service;* and *cost-benefit considerations*. The main categories and subcategories that emerged from the analysis of the interviews in this study in relation to the three levels of the strategic change model proposed by Ginter et al. [8] for managing and assimilating change are summarised in Table 1.

**Table 1:** Categories and subcategories for strategic change model

Main categories	Subcategories
***strategic thinking stage***	
Changes in the organization’s environment	Competitive nature of the field
	Developments in the market
	Customers’ and patients’ demand
***strategic planning stage***	
Instituting and assimilating changes	Embracing the change
	Digital transformation
	Various arenas in which digital change was adopted: equipment, customer service, administration, and management.
***strategic planning stage and strategic momentum stage***	
Obstacles in embracing change	operational difficulties
	learning the new technologies
	Information safety
	Blurring Personal and Professional Life Boundaries
	The generation gap
Added value obtained from embracing the change	Improving the quality
	Marketing advantage
	Improving Communication Quality among Dentists
***strategic momentum stage***	
*Quality of care and service*	
*Cost-benefit considerations*	*Time needed to train the staff*
	*Assimilating the new procedures*

### The strategic thinking stage

Examining the categories reveals that the first theme—changes in the dental clinic’s environment—correlate fully with the processes in the first stage of the model. As detailed above, at the stage of strategic thinking, the organization (in this case the dental clinic) analyses its environment and recognizes the importance of the change for better adaptation to its surroundings. The interviews conducted for this research indicate a consensus regarding the competitive nature of the field and the need to face unbalanced competition against large governmental organizations.

*We are aware of that… you keeps looking around. You don’t have to enter every competition*, *but you see what’s around him.*

*In large places like HMOs clinics … They advertise technologies*, *speak about service*, *the experts they have*, *things like that… I don’t really do that…*.

There is also consensus regarding the developments in the market, how digital equipment and methods are employed, and the working environments in which the clinics are active: equipment, technicians, and other service providers.

*I see it in every area—from appointment scheduling*, *digital appointments*, *digital x-ray scanning. I see it in the communication through WhatsApp*, *text messages*, *this whole area of internet aids*, *Google… everywhere*, *the labs have almost all been digitalized.*

*Technology advances much faster than medicine. They keep giving us new materials*,*new tools*, *new devices… It’s [happening] at a dizzying pace. Not a month goes by without devices update*, *devices improvement…*.

This stage requires a thorough inspection of the surrounding conditions, understanding of their developments, and an assessment of their consequences. When analyzing these environmental changes, the interviewees related to the patients as another element that demands change. This requires them to adjust and adapt so that they are able to stay in the competition and meet their patients’ demands.

*No doubt*, *from the patients’ perspective*, *there is the added element of branding*, *of visibility*, *however you want to call it. It’s perceived as advanced when you make it digital… This is why we must [change].*

Some doctors noted that they did not believe in certain technological advances, yet they feel pressured to adopt them, sometimes against their will, because of customers’ demand and the need to utilize advertising, marketing, and public relations in a competitive market.

*A patient sees this as standard nowadays. He wants to be shown on a screen… to be surrounded by digital*, *it gives him a sense of advancement*, *progress*, *although in my opinion this is all just for show*….

### The strategic planning stage

The second stage of the model is the strategic planning stage. This is when the first stage is translated into an action plan, comprising a series of steps the organization must take [8, 17]. In the analysis, the themes that correlated with this stage were found to be the following: instituting and assimilating changes, and Obstacles in embracing change. The sub-themes- embracing the change, digital transformation, and themes dealing with various arenas in which digital change was adopted, as detailed in Table 1. The interviews show that participants are familiar with digital transformation and embrace it to varying levels.

*Doctors are doing it… everything is computerized… the engraving too… implants with computerized navigation… There is actually a move from analog to digital dentistry.*

*In the past they would take measurements with all kinds of materials*,* today this is done with digitation.*

None of the interviewees reported a structured and thought-out strategy of change, with a plan, a budget, or division of labor. Yet it seems that in practice, they do embrace changes. For instance, interviewees indicated that they brought new technologies into their clinics. The sub-themes we identified point to several organizational levels where change is implemented: equipment, customer service, administration, and management.

Equipment: There seems to be consensus regarding the importance of the new technologies to medical care, patients’ safety (radiation, hazardous materials), clinical and aesthetic quality of care, and the possibility of visualizing the final outcome in treatments such as crowns or major dental restoration.

*In surgeries of jaw shifting*, *you can really see a computerized simulation of a skull. In the field of dental and bone implants*, * much of the design today is computerized.*

*Of course it’s all becoming digital. The x-rays*, *the record itself*, *basic software—and yes*, * also the typing in of the information*, *but also the fact that you have digital x-ray*, *it saves you time*,*it saves on radiation*, *it saves physical space—cabinets and archives.*

The dentists stated that these developments result in better dental care and more safety for patients.

*In dental restoration*, *the crowns*, *for example*, * are more accurate. They are more precise with digitation.*

*[Digital tools] can minimize risks for the patients*, *like hitting nerves*, *hitting blood vessels*, *putting it in a place that will also give aesthetic results and not just because there’s a bone there.*

Yet the dentists also indicated operational difficulties in learning the new technologies or handling technical problems that may arise when using digital equipment.

*You have to be very technical sometimes. This is one of the disadvantages.*

The interviewees also reported new technological tools used for running and organizing the clinic. These include technologies for storing information, creating and keeping medical records, or handling digital imaging. Yet participants reported that they did not rely exclusively on these new technologies and continued to simultaneously use the more traditional methods.

*You know*, *in my clinic*, *only if it burns down will the information disappear. But this way*, *when it’s in the computer*, *if I did or didn’t save*, *if there’s back up or there isn’t any*,*if hackers steal from you*, *come on. I use it*, *but I also keep what I’ve had so far.*

Interviewees also expressed concern relating to the link between technological advancements and issues of information safety, maintaining medical confidentiality, and protection against hacks and ransomware.

*It’s breached all over*, *in terms of information security too*, *there are many companies where hackers have accessibility to personal data; That’s another thing*, *this ransomware issue. It’s a bit disturbing that it’s breached*, *because you are exposed and can be hacked.*

According to interviewees, customer service and administration have also undergone digital transformation. For example, they reported using digital means for communication with customers, sending digital reminders, and scheduling appointments. Yet, despite prevalent use of such software in private clinics across various medical fields, in dentistry, it seems that scheduling appointments and managing patients and appointments is still not entirely computerized.

*The communication has become very accessible*, *well-documented. This greatly shortens timetables*, *and I think in the end*, *it improves the medical care.*

Here too interviewees reported that they were often dissatisfied with the changes.

*Some people can give their opinion over the phone or Zoom*, *but I don’t like that…*.

*There is the university*, *where everything is digitalized*, *and at my private clinic it’s a little less digital*, *meaning that I use old-fashioned methods … I’m a little scared of all that digitalization.*

Some of the interviewees were reluctant to use digital tools in customer service fearing that these devices might blur the boundaries between personal and professional life by causing them to work outside the official office hours.

*There are very few [people] who know how to separate things. They have two phones and separate business accounts*, * ncluding WhatsApp and everything. When they disconnect—this is it*, *you can’t go into that bubble. But you tell me*, *what doctor today has a bubble? The competition bends all the rules.*

The extent to which change was implemented in the clinics was evidently different across interviewees, and it seemed to depend on the age of the doctor/manager. Some of the interviewees claimed that they did not belong to the generation that embraces technological change, as opposed to younger doctors.

*The young generation*, *they have a very fast and steep learning curve compared to the old generation. So what we did*, *we just made it obligatory*,* all the students could only take digital images… these students know better [than advisors and older dentists]*, *and slowly convinced them to gradually move to digital.*

The interviewees also discussed the benefits of embracing new technologies and their added value. Beyond improving the quality of diagnosis and care, as mentioned above, interviewees also said that the new technologies were useful for marketing, as they added to the dentist’s reputation and the positioning of the clinic as advanced and safe. Some participants, however, said that this image was not necessarily true to reality, or that presenting the clinic as digitally innovative was not always relevant to the quality of its practitioners or the treatment they provided.

*For them it’s the number one marketing tool. A laser microscope*, *all these things*, *the Facebook and internet are for them platforms for marketing. In my opinion*, *these young digital guys make a very aggressive use of this.*

*There is an advantage to those who highlight their use of advanced technology because people look for it… people see computerized care all this rubbish that they advertise—people like that*, * I’m afraid. I want them to advertisev*, *but not just that.*

Another added value that participants reported related to the convenience and quality of the communication between dentists within the same clinic or with colleagues outside the clinic. According to the interviews, digital technologies facilitate smooth and steady communication and provide a shared platform for consultation and collaboration between dentists. This is useful, for instance, when several experts are needed to perform complex dental work. In addition, technologies that document and store medical information allow collaboration and assure the care continuity across the entire Israeli health system.

*I am a representative for… usually I have to travel abroad a few times a year*, *Instead… dentists and experts from the whole world*, *we shared information*, *asked questions*, *and showed presentations.*

*You can consult simultaneously with five doctors. In that respect*,* the possibilities are endless.*

In the category of *obstacles in embracing change*, many participants stated that despite the consensus about the importance of innovative medical equipment such as scanners, they found it difficult to institute changes in the administration and customer service aspects of the clinic.

*For a person who is originally analog*, *I am forced to learn digitalization. I need it for the hospital… everything there has become digital. But in my clinic*, *if I have to choose what and how to work- I still choose no digitation*.

Some interviewees indicated that even though they also worked in hospitals and HMOs, where they were required to use digital technologies, in their own clinics they had free choice, and they did not rush into going digital in administration and service.

*I do it when I have to*, *but in my own court*,* where I set the rules*, *I take my time. It takes me longer to do things digitally. I do it at a slower pace.*

According to interviewees, their reluctance to go digital derived from a set of difficulties and obstacles both in purchasing the technologies and in assimilating them in daily use. These obstacles included difficulty in training workers to use new systems, concern about keeping patients’ information private, fear of losing information due to problematic storage, securing backup, and dealing with cyber attacks. Most dentists stated that they kept duplicate records and even avoided rapid embrace of new systems to prevent these complications.

*I’m not afraid of using [technology] but hackers or all kinds of people can take information that I wouldn’t want to give them*, *and I have to keep all imaging for seven years*, *so I keep it for myself. I use digitalization for communication*, *but nothing can really replace pen and paper.*

### The strategic momentum

The final stage of the strategic model involves executing the plan over time, evaluating and providing feedback on actions taken, and coordinating changes with the internal and external environments of the clinics. During this stage, there is a circular process that overlaps with the second stage of planning. The categories related to the added value of embracing the change belong to this stage, but they are also included in the planning stage in this analysis. Several relevant categories emerged from the interviews- *Quality of care and service* and *Cost-benefit considerations in embracing change*.

When discussing Cost-benefit considerations, most of the interviewees stressed the differences between their clinics and the large medical centers that are often affiliated with HMOs or other big organizations. They explained that because private clinics have lower revenues and smaller crews, investing in advanced equipment and staff training is a big financial burden for them, which is not always justified in terms of return on investment.

*There’s also the element of price. If you buy a scanner now*, *no doubt*, *it improves your image*, *also somewhat your work etc.… But prices are high. If you want to get yourself now a panoramic device*,*a C.T.*, *we’re talking hundreds of thousands of shekels… This has financial implications.*

Two sub-themes in the category of cost-benefit were the *time needed to train the staff* and *assimilating the new procedures*. These are especially problematic in small-scale clinics, where the staff is small. In such cases, it is unrealistic to shut down all activity due to lengthy employee training. Most interviewees indicated that this consideration causes small clinics to lose their competitive edge against the big or corporation-owned clinics.

*Every new technology requires times. There’s a learning curve. For example*,*the microscope we’re using—it’s about six months to a year of learning how to work with this tool. It took me—I’m not exaggerating—must have taken at least a year to learn how to read a C.T. properly*, *to learn how to deal with this software. It just took a long time to learn that.*

All interviewees believed that building a reputation as being cutting edge carried important value to the dentists and clinic, and so did using digital advertising on internet platforms and social networks.

None of the doctors, however, reported conducting an objective appraisal of the return gained from their advertising efforts. Moreover, the absence of structured plans to integrate new systems was also noted in the findings.

## Discussion

The findings from the current research show that far-reaching changes occur in the market of private dental clinics in response to local and global developments, as well as changes in the governmental healthcare market. It is evident that strategic adaptation and change, including digital transformation, are essential for survival in this highly competitive market, which is increasingly leaning towards a more public-centric approach in many aspects of dentistry [4, 8].

Examining the theoretical framework to characterize these processes reveals that the stages of the model apply inconsistently, showing high level of variability. In stage 1, we obtained almost full consensus regarding analyzing the environment and understanding the competition within the dental market. The findings suggest that participants have a strong understanding of competition in the dental market, the need for digital transformation, and customer needs and demands. This highlights their skill in assessing industry changes and their competitive impact. It highlights the need for adaptation and relevance to thrive and survive.

However, in the following stages, the findings point to different perceptions regarding how to translate the insights obtained in stage 1 into practical steps. For example, at the strategic planning stage, in which the first stage is translated into an action plan—i.e., a set of steps the organization must take, including designing personal objectives [8, 17]—the evidence points to varying levels of activity conducted to implement changes, both in clinical and service aspects. These activities do not take the form of an organized and planned process that involves key players in the clinic or offers distinct perspectives in order to guarantee critical thinking and creative problem solving [18].

The research suggests that the dental clinics lacked a systematic approach to incorporate new technology and new systems into the dental clinic. This absence of structured planning in adopting change processes becomes evident when examining the gap between the theoretical model and actual practice, especially when comparing stages of planning and implementation. Such an oversight might not be beneficial for clinics when benchmarked against standard models of change implementation. This can adversely affect the clinic’s operational efficiency, leading to poor adoption rates and difficulties in effectively implementing new technologies, resource wastage and a potential decline in the quality of care provided.

The findings indicate that the clinics diverged on implementing the new technologies. The distinctions between the clinics in embracing change reflect the different nature of the clinic manager, his or her worldview, and tendency to embrace or reject technology, which is often dependent on age. These findings correlate with those of previous research, which have indicated that adopting new technology is tied to generational status and technological inclination [44].

Difference in embracing change was also detected in relation to the area of change. Changes in equipment or materials, for example, were seen as necessary both medically and for the clinic’s reputation. The doctors’ beliefs about how customers perceive these developments lead them to take a customer-orientated approach, believing that it may enhance customer satisfaction as well as clinical care, as shown in previous research [29]. Such changes are sometimes implemented against the professional opinion of the dentists, who perceive them as focused on image yet lacking in true clinical value.

As opposed to clinical equipment, in the areas of service and administration, digital transformation is not fully implemented. Evidently, the medical/ dental staff believe in its importance, yet some customers have objections. Overall, customers do not show clear preference for using digital over traditional methods such as phone calls and personalized customer relations, still being somewhat suspicious towards digitalized service. This is added to the difficulty of training employees that are sometimes reluctant to learn a new system. Additional difficulties have to do with ethical considerations relating to the patient-therapist relationship, exposure to extensive medical information, remote diagnosis and care, and other parameters that change the nature of that relationship [52]. In some cases, the process of digitalization is perceived as unnecessary and unhelpful for promoting the goals of the clinic. The major reasons for that are technical obstacles, the need to train the staff and to change work protocols, concern about exposing confidential information, and the risk of information theft and ransomware [52]. This leads to differences in the extent to which technologies are integrated into the clinic and workers are involved in the change. For example, decision making is usually done exclusively by the head dentist (who is often also the manager) and excludes the other employees, making the change more difficult to implement [8].

The next stage in the model, that of strategic momentum, includes repeated evaluation and feedback for steps taken to implement the change against internal and external environments. When examining this stage, we observe that the process of assessment and evaluation is performed to a limited degree and in an unstructured manner, usually not involving every staff member. We can see that all the interviewees acknowledged the added value of the change, recognized its obstacles, and considered its cost versus its value. But The findings suggest that none of the doctors conducted an objective appraisal of the return gained from the change processes. A lack of objective evaluation of the change processes can lead to suboptimal allocation of resources, and ultimately, underperformance. Without a thorough assessment of the return on investment (ROI) they may continue to invest in strategies that are not delivering the desired outcomes. Therefore, the absence of objective appraisals and structured plans can be detrimental to the overall performance, resulting in decreased quality of care, suboptimal financial outcomes, and inefficient use of resources. It is important to take a more rigorous and analytical approach to evaluate the effectiveness of their strategies and plans to integrate new systems effectively.

Here too, in this stage in the model, we see different approaches from different dental clinics, deriving from the substantial investment involved in the process. According to interviewees, digitalization is easier for large clinics (often a part of HMOs or other public sectors) with a big pool of patients, where faster return on investment is guaranteed and the process is thus more justifiable. This is in accord with studies that indicated cost as one of the most significant barriers to digitalization in dentistry [53]. Most interviewees did not mention designing strategic plans that included embracing new technologies. This may be accounted for by the fact that most of the participating clinics are run by dentists who do not necessarily have education in business management. The absence of structured strategic planning makes running the clinics and implementing change more difficult [54]. Since they often compete with institutional clinics affiliated with big healthcare organizations such as hospitals and HMOs (sometimes a part of the public sector), the lack of organizational planning is a weak point for the small private clinics. They are further challenged by the combination of limited financial resources and a small volume of activity. This combination of factors can make it difficult for them to achieve their goals or maintain their operations effectively; moreover, embrace change and invest resources in this process, especially in comparison to the growing public sector.

These processes are expected to intensify with the advancement of technology, imposing far-reaching implications on the activity of both private clinics and the health system as a whole [55]. As a result, the gap between the private sector and other oral health care systems, including the public sector, is expected to widen by the day.

### Limitations of the study

This study set out to explore the strategic implications of technology within dental practices. However, it was recognised that the analysis was limited by the range of perspectives included. The lack of a wide range of perspectives from different stakeholders - particularly patients and healthcare professionals from different specialties - has resulted in what can be considered a partial understanding of the complexities and nuances of technology integration in healthcare.

Furthermore, the design of the study did not include a longitudinal approach, which is crucial to capture the ongoing and rapid evolution of technology in healthcare. This omission means that the study may not have fully captured ongoing innovations and their lasting effects on healthcare practices and outcomes. This limitation underscores the critical need for future research to integrate multiple stakeholder perspectives and employ longitudinal study designs. Such methods are essential to ensure a holistic and up-to-date understanding of how technological advances are revolutionizing healthcare.

The sample of 20 dentists, mainly from central Israel, may not fully represent the broader attitudes of Israeli dentists toward technology integration in healthcare. While the specialist ratio reflects national averages, the study’s narrow geographic and professional focus could introduce bias. Additionally, the number of participants (20) might be considered small, given that private dental practices still provide the majority of dental services in Israel, particularly in adult care. While qualitative studies are often based on small samples, future research could benefit from expanding the sample size and including a wider range of geographic and professional perspectives. This would ensure a more comprehensive understanding of the strategic challenges facing private dental practices in the current competitive market.

## Conclusions

The findings of this study indicate that digital strategies are only partially implemented due to a lack of cohesive, long-term strategic direction. Participants excel in the first stage—strategic thinking—by being aware of industry competition, the need for digital transformation, and customer demands, demonstrating their ability to assess changes and competitive impacts. However, subsequent stages, such as planning and evaluation, are poorly executed or sometimes entirely absent. These stages often lack a structured approach that includes key stakeholders, resulting in a significant gap between recognizing the need for change and taking practical steps toward comprehensive transformation [4, 56]. This might not be to their benefit while competing and trying to survive in a market leaning towards a more public sector, creating already unequal competition.

The importance of this study lies in its deep understanding of the challenges faced by private dental clinics and the broader implications for the dental market in Israel. By identifying these barriers and analyzing their impact, the research provides valuable insights for developing effective strategies that can enhance the quality of care, ensure long-term sustainability, and maintain a competitive edge in the rapidly evolving dental healthcare landscape.

## Data Availability

The datasets used and/or analysed during the current study available from the corresponding author on reasonable request and in accordance with the relevant guidelines and regulations of the Ethics Committee.

## Abbreviations

HMO: Health Maintenance Organization
